# Do women with eating disorders who have social and flexibility difficulties really have autism? A case series

**DOI:** 10.1186/2040-2392-6-6

**Published:** 2015-05-13

**Authors:** Will Mandy, Kate Tchanturia

**Affiliations:** UCL, Research Department of Clinical, Educational and Health Psychology, Gower Street, London, WC1E 6BT UK; King’s College London, Psychological Medicine, Institute of Psychiatry, London, UK; Psychological Medicine Clinical Academic group, South London and Maudsley NHS Trust, London, UK; Illia State University, Tbilisi, Georgia

**Keywords:** Eating disorders, anorexia nervosa, Autism spectrum disorder (ASD), gender differences, Autism Diagnostic Observation Schedule (ADOS), clinical interview

## Abstract

**Background:**

Many women with eating disorders (EDs) have social impairments and difficulties with flexibility. It is unclear to what extent these are manifestations of an underlying autism spectrum disorder (ASD); or whether they are instead the consequence of starvation, anxiety, low mood or obsessive compulsive disorder, all of which are highly prevalent in EDs. The resolution of this clinically and theoretically important uncertainty will require the use of gold-standard ASD assessment measures. To date these have not been employed in ED research. This case series is the first report of a well-validated, direct-observational measure of ASD, the Autism Diagnostic Observation Schedule (ADOS), being administered to women with EDs. We aimed to learn about the feasibility of the ADOS in this population, and to contribute to debates about whether a sub-group with EDs really have ASD.

**Methods:**

Ten women (mean age = 26.4 years, range = 19 to 38 years) who had a suspected ASD due to social and flexibility difficulties and were receiving treatment for ED (seven anorexia, two ED not otherwise specified, one bulimia) at a specialist service (four inpatient, six outpatient) received an ADOS Module 4 assessment.

**Results:**

All 10 participants completed all activities of the ADOS Module 4. Five scored in the ASD range on the ADOS diagnostic algorithm. An additional two were judged likely to have ASD, even though they scored below the ADOS’s diagnostic threshold. This was on the basis of clinical observation, participant self-report and parent report. The seven women who we estimated to have ASD all reported autistic difficulties prior to the onset of their ED. They commonly described longstanding non-autistic neurodevelopmental problems, including dyslexia, dyspraxia and epilepsy. Only one had a childhood diagnosis of ASD.

**Conclusions:**

A substantial proportion of women with EDs who present with social and flexibility difficulties may have an unrecognised ASD, indicated by a constellation of autistic difficulties that appears to predate the onset of their eating problems. The ADOS is a useful component of an ASD assessment for adult women with ED.

## Background

Eating disorders (EDs) are characterised by markedly abnormal attitudes to body weight and food intake, which result in disturbed patterns of eating and behaviour. They include anorexia nervosa (hereafter ‘anorexia’), bulimia nervosa (‘bulimia’) and eating disorders not otherwise specified (EDNOS) [1]. Anorexia is diagnosed when a person becomes substantially underweight due to restricted eating, arising from a fear of putting on weight and a distorted body image [1]. Bulimia is defined as recurrent episodes of binge eating followed by inappropriate compensatory behaviour (for example, vomiting, laxative use, excessive exercise) in order to prevent weight gain [1]. EDNOS describes eating difficulties that are clinically severe, but which do not fulfil criteria for a specified ED such as anorexia or bulimia. Autism spectrum disorder (ASD) is a neurodevelopmental condition, characterised by pervasive difficulties with social reciprocity, social communication and flexibility [1].

At first glance, EDs and ASD would appear to have little in common. ASD is a disorder of social function and flexibility, which manifests in the first year of life. By contrast, bulimia and anorexia are concerned with abnormal eating behaviour, with typical onset around adolescence and early adulthood [2]. Males are at greater risk of ASD than females, whereas EDs shows the converse gender ratio, affecting 10 females for every male [3, 4]. While one-third of people with ASD have an intellectual disability, EDs are not related to intellectual impairment; and anorexia may actually be associated with above average intelligence [5, 6]. ASD is a lifelong condition; bulimia and anorexia fluctuate across the lifespan [7].

Despite this, there is currently interest among clinicians and researchers in the overlap between ED and ASD, with a specific focus on the hypothesis that ASD places women at high risk of developing anorexia (for example, [8]). This idea stems from Christopher Gillberg [9] and his collaborators’ [10, 11] observation that many women with anorexia show inflexibility and impaired social function, and that this may reflect the presence of an underlying ASD.

Gillberg and colleagues have published evidence to support their proposal that ASD and anorexia are associated, based on findings from two samples. They conducted a long-running study of a cohort of people (94% female) with adolescent-onset anorexia in Gothenburg, Sweden, estimating ASD prevalence in this group to be between 8% and 37% (see review by Huke and colleagues [12] for a synthesis of this work). Subsequently, the Gothenburg researchers reproduced their Swedish findings in the UK, reporting that seven of 30 women (23%) attending specialist ED clinics in London met criteria for ASD [13]. In addition to replicating previous findings in a new sample, this study is important because it extended the observation of high prevalence of ASD to an ED sample that included women with bulimia as well as anorexia. It should be recognised that the rates of ASD reported by the Gothenburg team, found in almost entirely female samples, are strikingly high, as in the general population of females ASD prevalence is estimated to be 0.3% [14].

In support of these diagnostic findings are studies of sub-threshold ASD symptomatology (‘autistic traits’) and of autistic cognition in anorexia. Investigations of autistic traits, measured by self-report using the Autism Quotient (AQ) [15], have shown elevated levels of autistic symptomatology in females with anorexia compared to controls [16–18]. Furthermore, at the group level, people with anorexia tend to show a cognitive profile characterised by poor theory of mind [19], cognitive inflexibility [20] and detail focused processing [21]. This mirrors the profile of cognitive difficulties found in groups of people with ASD (for example, [22]).

Nevertheless, some researchers have dismissed outright the idea that ASD is highly prevalent among people with ED [23]. Their scepticism arises from some substantial methodological limitations of the ASD-in-ED literature. One criticism is that all the ASD diagnostic findings in adults with ED come from one group of researchers, and that five of their six relevant studies are based on the same Swedish community sample [12].

A more profound challenge to the argument that many women with ED have ASD is that their social difficulties and inflexibility may not be truly autistic in origin. People with ED experience high rates of obsessive compulsive disorder (OCD), anxiety, depression and starvation, all of which can give rise to social impairment and rigidity that could be mistaken for symptoms of autism. The high rates of ASD reported in anorexia may reflect a problem of construct validity, with social and flexibility difficulties being mislabelled as autistic symptoms, rather than a true overlap between the two disorders [23].

One strategy for teasing apart autistic and non-autistic symptomatology is to use gold-standard, well validated assessment measures, designed to implement DSM/ICD accounts of ASD [24]. Such measures have not so far been used to assess adults with ED. In the initial Gothenburg anorexia studies, information was gathered in a general clinical interview, and this was then used to estimate ASD diagnosis [10, 25]. In later Gothenburg studies [11, 26, 27] general clinical interviews were supplemented by a structured Asperger’s assessment, the Asperger’s Syndrome Diagnostic Interview (ASDI) [28]. While the ASDI has much promise as a measure of autistic symptoms in high-functioning individuals, ASD prevalence rates based on its use are difficult to interpret for the following reasons. First, as its name suggests, the ASDI was designed specifically for the assessment of Asperger’s syndrome, and so it may not be suited to assessing the full range of presentations encompassed by the ASD diagnostic category. Second, it implements ‘Gillberg and Gillberg’ criteria for Asperger’s [29], which do not overlap fully with the almost universally accepted DSM and ICD accounts of ASD. Third, only preliminary information is currently available on the validity of the ASDI with respect to independent clinician diagnosis [28]. Fourth, in the ASD-in-ED studies, the ASDI appears to have been administered using a mixture of parent report, self-report and clinician observation, whereas it was designed and validated as an informant report interview [28]. A further complication when interpreting prevalence rates of ASD in anorexia is that multiple different definitions of ASD have been used throughout the Gothenburg studies, based on DSM-III, DSM-III-R, DSM-IV, ICD-10 and ‘Gillberg and Gillberg’ criteria [12]. These methodological factors may partly account for the wide variation of published ASD prevalence estimates in the Gothenburg sample (8% to 37%) [12].

The failure to use non-standardised assessments designed to implement a universally recognised (that is, DSM and ICD) definition of ASD may have led to over-estimation of the rates of ASD in ED [23]. The one study that has used a standardised, DSM-based ASD assessment, the Dimensional, Developmental and Diagnostic Interview - short version (3Di-sv) [30, 31], in young people with a restrictive ED did not find any evidence for high rates of ASD compared to controls [32]. This could indicate that previous findings of Gillberg and colleagues in adults with ED are an artefact arising from the use of non-standardised assessment of ASD; or it could have instead arisen from the nature of the participants studied, who were children and adolescents with ‘early onset ED’ [32], not adults who all met criteria for anorexia, as in the Gothenburg studies.

Thus, despite the influential work of the Gothenburg anorexia researchers (for example, [25, 10, 13]), it is currently unclear whether the social difficulties and inflexibility observed in adolescents and adults with EDs reflect underlying autistic difficulties; or whether they arise instead for other reasons, such as the effects of starvation, anxiety, low mood and OCD. A crucial step towards resolving this uncertainty will be the use of well-validated and standardised measures of ASD in samples of adults with ED. While parent report is a cornerstone of ASD assessment in children, in clinical practice with adults it is often difficult to attain, especially for those whose psychopathology may both reflect and cause family discord [33]. Thus we sought to pilot the use of a direct observational measure of ASD symptoms and diagnosis, called the Autism Diagnostic Observation Schedule (ADOS) [34].

The ADOS is the gold-standard structured observation tool for diagnosing ASD [24]. It is widely used in clinical and research practice, and can be administered with adults and adolescents as well as children. Despite this, we know of no reports of its use in adults with ED. We used the ADOS to conduct a series of clinical assessments of women with ED (anorexia, bulimia or EDNOS) whose social and/or flexibility difficulties had lead their clinical care team to suspect they had ASD. While the ADOS has shown excellent sensitivity and specificity in distinguishing adults with ASD from clinical controls, in common with all other assessments of autistic symptoms it was validated in a largely male sample [34]. Anecdotal reports and some indirect empirical evidence suggest that it may lack sensitivity for some symptoms of ASD as they present in high-functioning women [35]. As most people with ED are adult females with average or above IQ, we argue that the value of the ADOS as an assessment in this population cannot be assumed, making a pilot study necessary.

By offering in-depth qualitative and quantitative information about the use of this standardised direct observational assessment in clinical practice, we aimed to contribute to debates about whether the social and flexibility difficulties seen in some people with anorexia, and with ED more generally, are autistic in origin; or whether they are non-autistic difficulties that only superficially resemble ASD. By writing this case series, we also wished to present information about the feasibility of the ADOS in this population, to inform decisions about its future use in research and clinical practice with people with severe ED.

## Methods

### Participants

To be included in this case series, patients had to meet the following inclusion criteria: (1) attending a specific specialist service in the UK for adults with an ED, either as an inpatient or outpatient; (2) receiving treatment for an ED diagnosed according to DSM-IV criteria [36]; (3) has been identified by clinical care teams as having significant difficulties with social functioning and impairment, such that an ASD is suspected; (4) speaks fluent English. Ethical permission for the case series was obtained from the Clinical and Academic Group at the institution where this project was based. As all the information presented in this report was gathered to inform clinical care, rather than primarily for research, our study is a case-note review. Accordingly, we have sought to protect privacy and confidentiality by ensuring that data taken from clinical notes are not linked to any identifying information.

During the 2-month sampling frame of this study 11 individuals meeting the inclusion criteria for this case-note review were offered an ADOS as part of their clinical assessment. Of these 11, 10 decided to undergo an ADOS. Characteristics of this series of cases are shown in Table 1. All were women. The average age was 26.4 years (SD = 6.59).

**Table 1:** **Autism Diagnostic Observation Schedule (ADOS) Module 4 scores for women with an eating disorder and difficulties with social function and flexibility (numbers presented in bold typeface denote scores above ADOS threshold for ASD)**

	Eating disorder diagnosis	Additional difficulties	Body mass index	Clinical setting	ADOS communication	ADOS reciprocal social interaction	ADOS imagination/creativity	Stereotyped behaviours and restricted interests	ADOS social communication total	ADOS classification
1	Anorexia - restrictive	Depression	11.5	Inpatient	**3**	**8**	1	1	**11**	Autism
2	Anorexia - restrictive	GAD	13.95	Inpatient	**4**	**8**	2	0	**12**	Autism
3	Anorexia - restrictive	Depression	13.8	Inpatient	0	**4**	2	0	4	Non-spectrum
4	Anorexia restrictive	OCD	14.8	Inpatient	1	3	1	1	4	Non-spectrum
5	Anorexia - restrictive		16.5	Outpatient	**3**	**7**	1	2	**10**	Autism
6	Eating disorder not otherwise specified	Bipolar affective disorder	18	Outpatient	1	**5**	0	1	6	Non-spectrum
7	Eating disorder not otherwise specified	Depression	29.5	Outpatient	1	2	1	1	3	Non-spectrum
8	Bulimia	Borderline personality disorder	24.9	Outpatient	1	**5**	0	2	6	Non-spectrum
9	Anorexia - restrictive		16.4	Outpatient	**3**	**4**	1	0	**7**	Autism spectrum
10	Anorexia - restrictive	Epilepsy	15	Outpatient	**2**	**6**	1	0	**7**	Autism spectrum

GAD = generalised anxiety disorder; OCD = obsessive compulsive disorder.

### Measures

Autistic symptoms and diagnosis were assessed using the Autism Diagnostic Observation Schedule - Generic (ADOS) [34]. This is the most widely-used and best-validated direct observational measure of ASD. The ADOS has four versions (‘modules’), which are selected according to the expressive language level of the person being assessed. In the current study we administered Module 4, which is for people with fluent language who are adolescents or older. The ADOS Module 4, like the other ADOS modules, is a scripted encounter, during which the assessor orchestrates a series of situations, to create a standardised setting for the observation of social skills, communication, flexibility and responses to sensory stimuli. It takes, on average, between 40 minutes and 1 hour to administer. Behaviour is scored according to a comprehensive and standardised coding frame. The ADOS has a diagnostic algorithm, which sums scores from a subset of coded items. In Module 4 of the ADOS the algorithm outputs scores for ‘Communication’, ‘Reciprocal Social Interaction’, ‘Imagination/Creativity’ and ‘Stereotyped Behaviours and Restricted Interests’. There is also a ‘Communication + Social Interaction Total’, which is the sum of the Communication and Reciprocal Social Interaction scores [34]. The algorithm uses scores from the Communication, Reciprocal Social Interaction and Communication + Social Interaction scales to assign ‘autism’, ‘autism spectrum’ and ‘non-spectrum’ classifications. The distinction between each of these three categories is made on the basis of symptom severity. Scores from the Stereotyped Behaviours and Restricted Interests scale are not used in the assignment of ADOS-Generic diagnosis, as it is concerned with low frequency behaviours (for example, rituals, complex mannerisms) that do not necessarily present in the limited time frame of an assessment session.

The ADOS Module 4 possesses good reliability, when administered by a well-trained and experienced user [34]. It was designed to distinguish cases of ASD from typical development and from other difficulties such as intellectual disability, anxiety disorder and attention deficit/hyperactivity disorder. For this purpose, it has demonstrable criterion validity with a sensitivity of 0.90 and specificity of 0.93 when tested against clinician diagnosis [34].

In two cases parent-report information on ASD symptoms was collected (please see results for rationale) using the 3Di-sv [31]. This highly-structured interview has excellent reliability (inter-rater and test-retest) and validity with respect to clinician ASD diagnosis.

### Procedure

Individuals who presented with possible autistic difficulties were asked if they would like to undertake an ADOS assessment, as part of their clinical care. All ADOSes were administered by WM, an experienced, research-reliable user of the instrument, and ADOS trainer. These assessments were conducted in the participant’s place of main clinical contact: for inpatients this was in a room attached to their ward, and for outpatients it was in a day centre they attended for treatment. ADOS sessions were scored immediately after they had finished. Information from the ADOS was fed back to the individual’s clinical care team, who in turn shared it with the participant.

## Results

### Feasibility

Of the 11 people offered an ADOS, one declined, saying that she was not able to do the assessment that day. She was very anxious about sitting down to do the ADOS, as she felt compelled to be upright and active in order to burn calories. All of the 10 people who chose to have an ADOS were able to complete all Module 4 activities. Three showed notable signs of anxiety at the start, but for all of them this appeared to abate as the session progressed. In each case, it was not judged that this anxiety interfered with their ability to undergo a valid ADOS. All ADOS assessments took between 45 minutes and 1 hour and 5 minutes.

### ADOS scores and classifications

**Figure 1:**
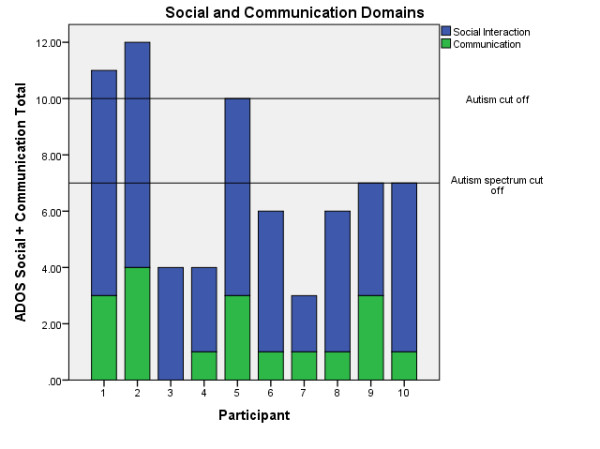
**Scores on the Autism Diagnostic Observation Schedule (ADOS) Module 4 Social and Communication Domains.**

While all 10 women in the current sample were perceived by their clinical care teams to have social and flexibility difficulties, they showed variability in their ADOS scores, as is illustrated in Figure 1. Three were classified on the ADOS algorithm as having autism, and a further two scored in the autism spectrum range. The remaining five scored below the ADOS’s ASD threshold.

### Qualitative descriptions of participants’ autistic characteristics, presented according to their categorisation on the ADOS algorithm

#### ADOS autism

The three women who received an ADOS autism classification showed a range of autistic symptoms in the assessment session. All three were coded as having unusual intonation, lack of descriptive and emphatic gesture, and unusual eye contact. All showed impaired empathy, and at times this was quite striking. For example, when commenting on a picture in a storybook of a male character who was clearly frightened, one participant described him as ‘excited’. Two showed markedly limited social insight. Two had compulsive ritualistic tendencies that were observable in the ADOS. For example, they both felt compelled to carefully and neatly arrange some pens that were randomly set out on the table.

All three people who scored in the autism range of the ADOS reported that their social difficulties were longstanding, and predated the onset of their ED. They all recalled serious social difficulties at school, whereby they struggled to make and maintain friendships. Two of the three also reported flexibility difficulties prior to their anorexia. For example one said she had always been ‘set in [her] ways’, while the other said she has been ‘obsessed by time and organisation’. The third described longstanding sensory hyper-reactivity. This included an aversion to strong smells, present since childhood, which made her feel revolted by many foods.

#### ADOS autism spectrum

The two women who scored in the ADOS autism spectrum range also presented with a constellation of social, communication and flexibility difficulties that appeared autistic in nature. While both were verbally fluent and articulate, they struggled with non-verbal aspects of communication such as gesture and the coordination of facial expression with verbal communication. Also, both scored on ADOS codes measuring social insight and empathy, indicating substantial and clinically meaningful difficulties with social function. For example, one participant had recently had to leave a job due to tensions with a senior colleague, and revealed a limited understanding of why these tensions had arisen, including how her behaviour might have contributed to them, or what she could have done to resolve the situation.

Reports from the women with autism spectrum classifications suggested that their difficulties were longstanding, and predated the onset of their ED. Neither appears to have ever had a close or sustained friendship, and their eating difficulties were preceded by painful experiences of social isolation and bullying at school. Both women also had a history of non-autistic neurodevelopmental difficulties. One had epilepsy, and both were reported to have been assessed by psychologists in childhood for dyslexia. Epilepsy [37] and dyslexia [38] are associated with ASD and, in the context of social difficulties, are an acknowledged indicator of the need for ASD assessment [39].

#### ADOS non-spectrum

Although P6 and P7 scored below the ADOS threshold for ASD, both may actually have ASD. P6 reported that in childhood she was diagnosed with ASD at a National Health Service (NHS) Child and Adolescent Mental Health Service, where she was referred after having been excluded from primary school, where she had significant friendship difficulties. She also reported that a female first-degree relative has Asperger’s syndrome, suggesting that she carries an elevated genetic risk for ASD [40]. She described having compensated for her social difficulties by observing and imitating other girls. For example, she said that she made a conscious effort to copy how others use gestures and make eye contact. It is interesting to note that she has some interests, such as fashion and veganism, which are not inherently unusual, but which she pursues with an unusual, and possibly autistic, intensity.

As we were uncertain about the validity of P6’s ADOS, we sought more information about her development by contacting her mother. We administered a structured, widely used parent-report interview, the 3Di-sv, over the telephone. This was done retrospectively, as we asked P6’s mother to give answers based on her daughter’s childhood (as opposed to present) behaviour. Overall, P6’s scores on the 3Di-sv were elevated compared to typically developing children, but were not in the ASD range. P6’s mother described her daughter in childhood as having a range of longstanding neurodevelopmental difficulties (dyspraxia, dyslexia), a strong tendency towards obsessional behaviour, very limited social relationships and trouble understanding body language.

P7 showed a range of social capacities in the structured, one-to-one context of the ADOS Module 4, which meant that she did not score highly on its diagnostic algorithm. In fact, as can be seen in Table 1, she got the lowest ADOS scores of any of the participants in this study. However, she gave several indications that she has a history of atypical neurodevelopment, and may actually have an ASD. She reported that in childhood and early adolescence she enjoyed flapping her hands and rocking her whole body back and forth, which is a classically autistic behaviour. She only stopped once she realised that this was considered odd by peers. Also, she recalled being a withdrawn child who preferred her own company to that of others, and said that in school, difficulties with attention and receptive language meant that she was often confused and uncertain about what she was supposed to be doing. It appears that these difficulties have persisted into adult life. P7 seems to be rather socially naive, and this has left her vulnerable to exploitation, as when she leant a large sum of money to her friend, who has since become difficult to contact and has not paid the money back. She also recounted that she often struggles to understand what to do when given instructions on her voluntary work placement, and is left with a dilemma about whether to ask repeated questions, which she knows can annoy people, or whether to give up trying to understand. Furthermore, P7 gave vivid examples of sensory processing difficulties, including finding it hard to function socially in groups (as opposed to one-to-one), as she can become overwhelmed by multiple voices.

Given uncertainty about P7’s ADOS score, we administered the 3Di-sv to her mother, asking for retrospective reports from childhood. P7 scored in the ASD range on this interview, with her mother reporting that P7 had wide variety of autistic symptoms in childhood. P7’s mother described a child who had been solitary, with little interest in other children and a strong need for control, who had a range of sensory and repetitive behaviours, and who struggled to understand what to do in school. She also reported that she (that is, P7’s mother) and P7’s brother have dyslexia.

Of the five participants not considered by the ADOS algorithm to have ASD, two presented as clear-cut non-ASD cases. These were participants 3 and 4 in Table 1. Both showed a range of strengths in the ADOS that would be inconsistent with an ASD diagnosis, such as skilfully coordinating verbal and non-verbal communication during back-and-forth conversational exchanges, making and responding to social overtures in a natural, spontaneous manner and generally fostering good rapport with the ADOS administrator. Nevertheless, both participants did have some subtle characteristics that could be understood as sub-clinical autistic traits. Both reported low levels of social motivation, even in childhood. For example, P3 said that she had few friendships during childhood, and that she had not wanted more. She spoke of valuing a capacity for silent companionship in her current friends. Also, both P3 and P4 described themselves as somewhat inflexible people.

P6 presented with both social difficulties and compulsive behaviour, although these were not classically autistic in quality. Rather, she reported an intense aversion to social contact, conveying that she found the idea of social intimacy intrusive and repulsive. She presented as being quite suspicious and withdrawn, and there are concerns within her clinical team that she may have emerging psychotic difficulties.

## Discussion

Among people with EDs, social difficulties [41, 42] and inflexibility [43] are common. It is currently unclear to what extent these are manifestations of an underlying ASD; or whether they are instead epiphenomena of ED which only superficially resemble autistic symptoms [23, 8]. A step towards resolving this clinically and theoretically important uncertainty will involve the use of gold-standard ASD assessment tools in adults with an ED. To this end, we report a case series of Autism Diagnostic Observation Schedule (ADOS) [34] assessments, carried out with 10 women who had an ED, as well as social and flexibility difficulties that had led their clinical care teams to suspect they have ASD. This is, to our knowledge, the first report in the scientific literature of the ADOS’s use in ED, and we aim to provide information about its feasibility in this population, as well as information about ASD in our sample.

Overall, our findings are supportive of the idea that there is a genuine overlap between ASD and ED [9, 10, 13]. Among the 10 women assessed, five met criteria for an autism spectrum disorder on the ADOS Module 4 algorithm. This reflects the fact that they demonstrated a range of social and communication difficulties that are consistent with the autistic phenotype, including unusual eye contact, limited empathy and social insight, abnormal use of gestures to compliment verbal communication and atypical intonation.

All five women scoring for ASD on the ADOS had anorexia and a body mass index below 17. One interpretation of this is that their high scores on the ADOS do not signify genuine autistic symptoms, but instead reflect the severity of their ED, including the effects of starvation. However, this possibility is countered by reports from each of these five women that their autistic difficulties had been present in childhood, prior to the onset of their ED. This is consistent with the fact that ASD is a developmental disorder that appears early in life [44], and counters the idea that their social and flexibility difficulties are merely a consequence of their eating and emotional difficulties.

Nevertheless, more work is required to test adequately the hypothesis that ASD is a causal risk factor for ED. To this end, we suggest several research strategies. First, prospective studies are needed to investigate whether in some cases ED is preceded by ASD or autistic traits. Second, research in this area should go beyond examining symptom overlap, to consider shared underlying mechanisms of ASD and ED. The current interest in endophenotypes for mental disorders, exemplified by the NIMH’s Research Domain Criteria (RDoC) strategy [45], can inform investigations into whether ASD and ED share common cognitive and neurobiological underpinnings. Third, family studies investigating rates of ASD in the pedigrees of people with ED, twin studies and genome-wide complex trait analysis could all be used to estimate the amount of shared genetic risk for ASD and ED.

We identified two women (P6 and P7) who did not score in the ASD range on the ADOS, but may actually have an autistic disorder. We suspect they have ASD for a number of reasons. First, they reported that in childhood they had various neurodevelopmental difficulties associated with ASD, including problems with sensory processing, emotion regulation, language and attention [46–48]. Second, both were referred in childhood to psychologists for neurodevelopmental difficulties, and one actually received an ASD diagnosis at this time. Third, they described histories of childhood social difficulties, leading to social isolation and bullying which are consistent with having ASD [49]. Fourth, they had family members with substantial neurodevelopmental difficulties, including Asperger’s syndrome. Family members of people with ASD are at substantially increased risk of ASD and other neurodevelopmental problems [50]. Fifth, when we contacted the mothers of these women to learn more about their development, they reported a range of childhood difficulties consistent with ASD, including non-social aspects of ASD such as inflexibility and sensory abnormalities. P7 scored above the 3Di-sv thresholds for an ASD diagnosis; and P6 scored below it, but nevertheless showed elevated parent-reported levels of autistic traits.

The assessment of ASD in females with normal-range IQ is notoriously difficult, as there is a female autistic phenotype, characterised by subtler difficulties compared to equivalent males [3, 51, 52]. This may help us understand why P6 and P7 did not score in the autistic range on the ADOS. Studies of ASD in women have suggested that compared to men, women tend to score lower on the ADOS Module 4 [52]. This likely reflects a key element of the female ASD phenotype, which is a capacity for camouflaging autistic characteristics in social interactions [35]. In this context, camouflaging denotes an effortful, conscious masking of underlying autistic difficulties, using imitation, reasoning and symptom suppression [52]. It is notable that both P6 and P7 gave examples of how they have deliberately camouflaged their autistic difficulties. For example, P7 spoke about suppressing the urge to rock and flap, and P6 described how she consciously learnt how to use eye contact and gesture by imitating other girls. These findings highlight the challenges of assessing ASD in women with EDs, showing that while the ADOS is likely a useful tool in this population, as in any ASD assessment, its algorithm should never be used as the only basis on which to make a diagnostic decision.

Our findings must be considered in the light of several limitations of the current study. While the ADOS is well-designed to distinguish between autistic and non-autistic psychopathology (34), it is nevertheless possible that we have overestimated the levels of autistic symptoms in our sample, due to the presence of starvation, anxiety, depression, attention deficit/hyperactivity disorder and other difficulties that co-occur with ED at above-chance levels. One way to mitigate this problem in future would be to include a control group of women matched for ED severity and co-occurring conditions, but who do not have marked social and flexibility difficulties. Another strategy for increasing the validity of our findings would be to conduct a multi-modal assessment for all people in the study, including a standardised informant-report developmental history, and to use the resultant information to derive a clinician consensus diagnosis based on DSM-5 criteria. Any study seeking to estimate the true prevalence of ASD in ED should take this approach.

## Conclusions

The findings we present come from a pilot case series, and must be considered preliminary. Nevertheless we believe they justify the following recommendations. First, we found some evidence for inadequate assessment of ASD in females, and action is required to redress this. In our study of 10 women with an ED, five scored up for ASD on the ADOS and a further two did not reach ADOS thresholds, but we nevertheless suspect that they have ASD. But of these seven women, only one had a prior autistic diagnosis. All of them reported significant social impairments in childhood before the onset of their ED, often accompanied by peer victimisation; most had histories of serious neurodevelopmental difficulties, such as dyslexia and epilepsy; and longstanding difficulties with sensory processing and flexibility were commonly reported. Several had been assessed as children by mental health professionals. And yet their ASD had been missed in childhood, and as a consequence they did not receive appropriate support and understanding at this time. These specific experiences fit with a broader picture of females missing out on ASD diagnoses and services. Females with ASD are under-represented in ASD clinics [53]. When they are identified, this tends to be later than for males [54] and they require more severe symptomatology than males to be meet ASD diagnostic criteria [55]. Urgent efforts are required to elucidate the characteristics of the female autistic phenotype and to use this knowledge to reduce the current diagnostic bias against girls and women with ASD.

A second recommendation is that the ADOS is a potentially feasible and valid tool for assessing ASD in women with an ED. We found it could be administered according to protocol even with people suffering from acute and severe anorexia. We have presented qualitative evidence that the ADOS provided valuable information for diagnosing ASD; but also that scores on its diagnostic algorithm are not in any sense definitive. Our experiences suggest that, wherever possible, observations made during the ADOS should be supplemented by reports from informants, including other clinical staff and family members, and this information should be used to inform a consensus diagnosis reached between clinicians [39].

A third recommendation is that research is required to provide clinically useful information about people who have ED and ASD. It will be important to discover the true prevalence of ASD among people with anorexia, if rates of ASD differ according to ED diagnosis, and whether individuals with ED and ASD have a distinct aetiology, treatment needs and prognosis compared to people with ED who do not have ASD. We think that further research in this direction will help us to develop more precisely targeted interventions; and can also provide information on the female phenotype of ASD.

## Abbreviations

3Di-sv: Short version of the Dimensional Developmental and Diagnostic Interview
ADOS: Autism Diagnostic Observation Schedule
ASD: Autism Spectrum Disorder
ED: Eating Disorder
EDNOS: Eating Disorder Not Otherwise Specified
OCD: Obsessive Compulsive Disorder.
